# Influence of Cross-Linking Conditions on Drying Kinetics of Alginate Hydrogel

**DOI:** 10.3390/gels9010063

**Published:** 2023-01-12

**Authors:** Magdalena B. Łabowska, Maria Skrodzka, Hanna Sicińska, Izabela Michalak, Jerzy Detyna

**Affiliations:** 1 Department of Mechanics, Materials and Biomedical Engineering, Faculty of Mechanical Engineering, Wroclaw University of Science and Technology, 50-372 Wroclaw, Poland; 2 Department of Advanced Material Technologies, Faculty of Chemistry, Wroclaw University of Science and Technology, 50-372 Wroclaw, Poland

**Keywords:** sodium alginate, cross-linking, hydrogel, shrinkage, dimension changes

## Abstract

Hydrogels are three-dimensional cross-linked polymeric networks capable of a large amount of fluid retention in their structure. Hydrogel outputs manufactured using additive manufacturing technologies are exposed to water loss, which may change their original shape and dimensions. Therefore, the possibility of retaining water is important in such a structure. In this manuscript, kinetic analysis of water evaporation from sodium alginate-based hydrogels exposed to different environmental conditions such as different temperatures (7 and 23 °C) and ambient humidity (45, 50 and 95%) has been carried out. The influence of the cross-linking method (different calcium chloride concentration—0.05, 0.1 and 0.5 M) of sodium alginate and cross-linking time on the water loss was also considered. Studies have shown that a decrease in the temperature and increase in the storage humidity can have a positive effect on the water retention in the structure. The storage conditions that led to the least weight and volume loss were T 7 °C and 95% humidity. These experiments may help in selecting the appropriate hydrogel preparation method for future applications, as well as their storage conditions for minimum water loss and, consequently, the least change in dimensions and shape.

## 1. Introduction

Hydrogels are soft materials, consisting of three-dimensional networks of polymer chains linked to each other and embedded in an aqueous solvent. Strong connections of polymer chains formed during the cross-linking process make them insoluble in water. However, the presence of hydrophilic functional groups in their backbone results in a significant amount of fluid absorption. Due to the hydrogels’ structure, they take an intermediate state between liquid and solid [1,2,3]. The amount of fluid contained in the structure must consist of at least 10% of the total volume. On the other hand, the maximum fluid content depends on polymer concentration, cross-linking process, etc., but this quantity can change over time as a result of the drying process. Water amount and its organization in the hydrogel structure can affect its final characteristic, such as mechanical properties (e.g., viscoelastic properties, strength, stiffness), likewise, properties connected with morphological features (e.g., porosity, permeability to oxygen). Consequently, by modification of the hydrogel manufacturing process, physicochemical properties can be adjusted for their applications [4,5,6].

**Figure 1:**
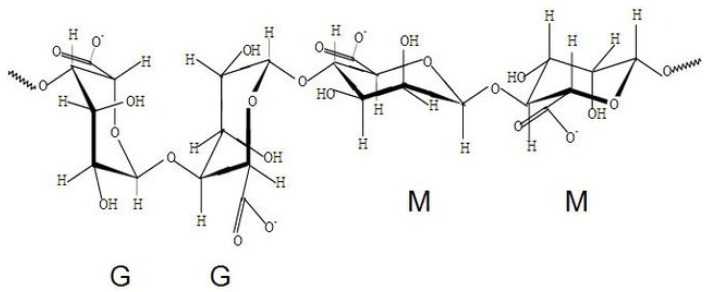
Molecular structure of alginate (M—β-D-mannuronate blocks, G—α-L-guluronate blocks) (Available via license: CC BY 3.0).

Hydrogel material is becoming increasingly interesting, and depending on the demand, it can be processed into different forms, e.g., moisture-absorbing beads in disposable baby diapers, in liquid form as a shower gel, as thickening substances in jams in the food industry, as creams in cosmetics, or protecting beds in agriculture; as well as be used in applications in water remediation and biofuel production [2,7,8]. A promising application area is also in biomedical engineering, due to biocompatibility, low immunogenicity and lack of toxicity of these materials. Their highly hydrated, soft structure is able to mimic the extracellular matrix (ECM) and provide a porous but mechanically durable scaffold for in vitro models intended for two-dimensional (2D) and three-dimensional (3D) cell culture [9]. Furthermore, hydrogels are also used as a material for wound dressing, providing high absorbency of wound fluid and accelerated wound healing; drug delivery systems, allowing controlled drug release and therefore better biodistribution; as well as for contact lenses, dental products and other applications [4,6,10,11]. In the biomedical field, both natural (e.g., alginate, hyaluronic acid (HA), gelatin, collagen, fibrin) and synthetic polymers (e.g., polyacrylamide (PAAm), polyethylene glycol (PEG), polyvinyl alcohol (PVA)) are used in hydrogel manufacturing. The first commercially used material was hydrophilic poly(2-hydroxyethyl) methacrylate (PHEMA), which in 1960 was used for lens fabrication [10,12]. However, the use of natural materials that are easily biodegradable and can also provide durability is becoming increasingly prevalent. One of the most commonly used polymers for hydrogel production is alginate, a natural polysaccharide, obtained from brown seaweeds. Its biomedical properties, cellular compatibility and mechanical properties make it suitable for tissue engineering applications [13,14,15,16]. The stability of alginate hydrogel depends on physical properties—the composition of β-D-mannuronate (M) and α-L-guluronate (G) blocks contained in the chain structure (Figure 1). Their sequence and arrangement determine the brittleness and elasticity, as well as degree of water evaporation enclosed in their structure [13]. Mechanical properties are also contingent upon bivalent and trivalent cations used in hydrogel cross-linking. Their quantity also affects the water content in hydrogel and hence the evaporation rate [15]. Hydrogel dehydration can lead to hydrogel shrinkage, resulting in dimensional, structural, and particularly, mechanical property changes. During the drying process, hydrogels lose their flexibility and become stiffer and more brittle [17,18].

The polymer hydrogels used in the medical field are often processed using additive manufacturing (AM) technology consisting of several methods such as extrusion-based, inkjet and laser printing techniques. A high level of automation and shape reproduction makes the use of AM technologies simpler and more affordable compared to conventional technologies [19,20,21]. The obtained printout is processed and cross-linked after extrusion. The possibility of personalization and the freedom of the manufactured shape are just some of the advantages offered by 3D bioprinting [19,20,21]. The ability to create complex and obtaining repeatable structures that cannot be obtained through traditional, conventional processes is the primary benefit of employing AM technologies. On the other hand, the biggest challenge during the use of semi-fluid materials, such as sodium alginate-based hydrogels, as ink, is to develop a method for the shape retention immediately after the manufacturing process. Hydrogels, due to their high water content tend to lose water over time. Consequently, they are prone to shrinkage and shape deformation as compared to the initial product. This process may influence scaffold–tissue association by pressure on healthy tissues in the vicinity or can generate structural disorders [22].

There are some examples of alginate-based hydrogel shrinkage and shape deformation in the literature. Vargas et al. [23] investigated the shrinkage of hydrogels based on sodium alginate that included drying of calcium alginate hydrogel at temperatures ranging from 40 to 70 °C. Lyn et al. [24] in their work presented drying kinetics of calcium alginate at 25 °C depending on the structural characteristic (guluronate block composition). Kaklamani et al. [14] carried out dehydration of alginate hydrogel through exposure to high temperatures (100–200 °C) and showed the anisotropic nature of this material. A lot of research is carried out in the field of freeze-drying or supercritical drying, which is responsible for complete exclusion of water without changes in shape to obtain a porous structure (sponge). This method can be realized using supercritical drying with CO_2_, acetone, or methanol, or at high temperatures with high pressure [25,26,27]. Researchers from Massachusetts Institute of Technology (MIT, Cambridge, MA, USA) have developed a method that prevents hydrogels from dehydrating. This technique is based on physical cross-linking, modification of the cured surface of elastomer with benzophenone, and then covalent cross-linking of polymeric networks. It will allow the creation of flexible matrices with long-term hydration, while maintaining an extremely robust interface. Examples of utilization of this material include inter alia contact lenses, artificial skin and flexible bioelectronics [28]. On the other hand, Gong et al. [29] have developed a method where hydrogel shrinkage is utilized to scaffold more compact dimensions in production. This technique enables the size reduction of a manufactured object, which enables the fabrication of thin and accurate prints that are not achievable by conventional technologies. However, there is still not much research on alginate hydrogel shrinkage, size and shape changes exposed to lower temperatures and depending on the concentration of the cross-linking agent, especially for preventing water loss.

The objective of this study was to evaluate the shrinkage of hydrogel spheres based on sodium alginate, as well as the deformation aptitude of alginate hydrogel cuboidal model (28 mm × 7 mm × 3 mm) in different environmental conditions. The effect of different concentrations of the cross-linking agent (0.05 M, 0.1 M and 0.5 M of calcium chloride), cross-linking time (10 min, 20 min and 40 min) and environmental parameters (temperature and humidity) on the mass and dimension changes of sodium alginate-based hydrogels was measured.

## 2. Materials and Methods

Alginic acid sodium salt (Sigma-Aldrich, St. Louis, MO, USA) was used for 2% alginate solution preparation. Calcium chloride nonahydrate (Avantor Performance Materials Poland S.A., Gliwice, Poland) was used for alginate cross-linking. Hydrogels were prepared as spherical samples for mass change investigation, and cuboidal models were used to examine their dimensional change. 

**Figure 2:**
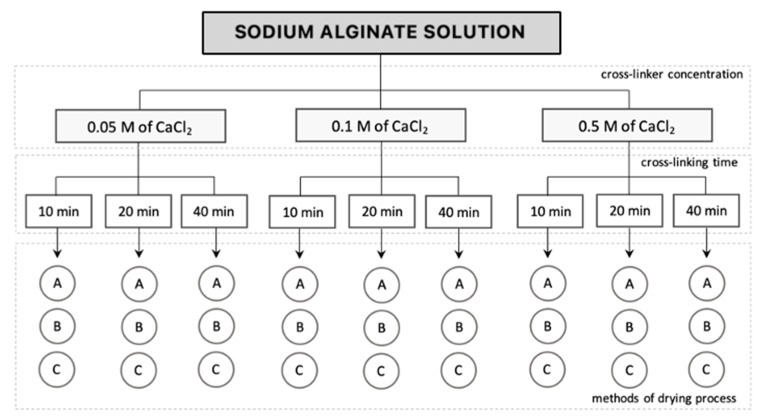
Layout of the hydrogel drying study including concentration of cross-linking agent, cross-linking time and drying method: A—at room temperature (23 °C) and 45% humidity, B—at 7 °C and 50% humidity, C—at 7 °C and 95% humidity (airtight container).

The alginate-based hydrogel beads were kept in three different environments for 3 h: (1) air drying at room temperature (23 °C) and 45% ambient humidity, (2) air drying at lower temperature (7 °C) and 50% humidity, and (3) temperature 7 °C and 95% humidity; the alginate-based hydrogel beads were stored in an airtight container. The mass of hydrogel samples was measured at given time intervals (0 min, 10 min, 30 min, 60 min, 90 min, 120 min and 180 min). The drying kinetics of the hydrogels were determined for different concentrations of the cross-linking agent (0.05 M, 0.1 M and 0.5 M of CaCl_2_) and cross-linking time (10 min, 20 min and 40 min). The general scheme of the conducted experiments is presented in Figure 2, and Table 1 shows the process parameters of all samples for both masses, as well as shape change studies.

### 2.1. Mass Changes of Alginate Hydrogels

To determine the kinetics of water loss from alginate hydrogels, samples were placed in different environments. The mass fluctuation was measured by balance (with a tolerance of 0.0001) every specified time interval: hydrogel after cross-linking (0 min), and then after 10 min, 30 min, 60 min, 90 min, 120 min and 180 min of drying. The research was carried out to determine the influence of the cross-linking conditions (time and CaCl_2_ concentration) and the hydrogel storage method on the water loss from its structure.

In order to compare the rate of water loss during a specific time interval, the following equation was used [30]:

(1) $$W_{L}\left(\%\right)=\frac{m_{0}-m_{end}}{m_{0}}\cdot 100\;$$

where *W_L_*—water loss, *m*_0_—initial alginate hydrogel beads mass, *m_end_*—final sample mass.

### 2.2. Dimension Changes of Alginate Hydrogels

Water loss from hydrogel is associated with its shape changes. Therefore, studies of the volume changes of a cuboidal hydrogel model of 28 mm × 7 mm × 3 mm dimensions in a time function have been carried out. The effect of the cross-linking conditions (time and CaCl_2_ concentration) was considered. 

In order to compare the rate of change in dimensions after drying time, the following equation was used [30]:

(2) $$D_{C}\left(\%\right)=\frac{V_{0}-V_{end}}{V_{0}}\cdot 100\;$$

where *D_C_*—dimensional changes, *V*_0_—initial alginate hydrogel beads volume, *V_end_*—final sample volume.

### 2.3. Statistical Analysis

Statistical analyses were carried out with Statistica software (ver. 13, TIBCO Software Inc., California, CA, USA). Comparisons of multiple independent groups were conducted using ANOVA tests. The Shapiro–Wilk test was performed (at a significance level of *p* ≥ 0.05) to assess the normal distribution of measurement data. Homogeneity of variance was also verified by Brown–Forsythe test (*p* ≥ 0.05). Determination of statistically significant differences (*p* < 0.05) between the research groups was conducted using a Tukey post-hoc test. For the other than normal distributions, non-parametric tests were performed. Determination of statistically significant differences (*p* < 0.05) between multiple independent samples was carried out using a Kruskal–Wallis test. 

Many statistical methods are available to analyze correlations between multivariate variables, including their significance for the final outcome. One such method is the PLS (partial least squares) analysis, a method that targets factor overcorrelation, which is associated with high response variability. A VIP (Variable Importance in Projection) indicator is used to rank the variables in order of importance [31]. It was used in this study to determine the contribution of variables at a significance level of *p* ≤ 0.05.

## 3. Results and Discussion

### 3.1. Additives to Alginate Ink Improving Mechanical Properties and Reducing Drying

The usage of sodium alginate-based hydrogel as ink in additive manufacturing technology requires the material to have such properties as to ensure uninterrupted and uniform transfer of ink from the nozzle to the print bed and also to maintain the appropriate shape of the printout, e.g., without collapsing. Additionally, during three-dimensional manufacturing, due to the drying process, hydrogel loses water, which results in a change in the target shape of the printout. One of the solutions to improve the rheological and mechanical properties, as well as minimize the effects of hydrogel drying, is to use additives of other polymers to alginate ink. Uniform extrusion of hydrogel from the nozzle is provided by low-weight sodium alginate, which after cross-linking exhibits low mechanical properties as a hydrogel [32]. The mechanical properties of biomaterials used in tissue engineering are crucial to provide support to the new tissue and space required for cell growth [33]. In order to improve the rheological and mechanical properties of alginate-based hydrogel, other substances such as derivatives of cellulose; usually in the form of nanofibers (CNF) or nanocrystals (CNC) [34,35]; or gelatin [36,37] or agar [38] are added to it.

The addition of derivatives of cellulose makes the hydrogel exhibit shear thinning behavior which facilitates the extrusion of ink providing better printability of the hydrogel [39,40]. At the same time, nanocellulose provides self-supporting properties and helps maintain printing precision and cross-linking speed [41], which allows the component to maintain its shape after fabrication. Derivatives of cellulose improve not only the rheological properties of the alginate ink but also the mechanical properties of the cross-linked hydrogel [40,41]. Increment of carboxymethyl cellulose (CMC) [39] or CNF [42] leads to higher Young’s modulus values. Due to these modifications, alginate ink can be used in applications that require repeatable printability and shape fidelity [39], such as in soft-tissue engineering [43] and regenerative medicine applications [42].

Alginate hydrogel with the addition of gelatin provides proper rheological properties during printing and improves mechanical properties [36]. Chung et al. [37], after mixing alginate with gelatin, obtained an ink with higher viscosity and storage modulus, while maintaining mechanical properties and similar cell growth performance. Giuseppe et al. [44], by increasing the gelatin content in the hydrogel, increased its compressive strength, making the material more durable. At the same time, as the gelatin concentration in the ink increases, its viscosity increases too. Therefore, it is crucial to find the proper proportions of alginate and gelatin. Increasing proportions of alginate in a mixture of gelatin leads to spreading upon deposition, while a high gelatin concentration leads to obstructed extrusion process due to increased viscosity [45]. Nevertheless, despite tunable mechanical properties and good printability, alginate–gelatin hydrogels degrade quickly when maintained at physiological temperatures, limiting their in vivo application [44]. Therefore, it is important to conduct further work on this biomaterial to allow for its wider use.

Agar content in hydrogel increases the viscosity of ink and guarantees better printing precision [38]. The addition of agar not only influences the rheological properties of the material, but also improves its mechanical properties such as stiffness and tensile strength [46]. Hydrogel that is prepared in this way fulfills the requirements of load-bearing tissue substitutes [46]. It has been verified that even a small amount of agar offers higher mechanical resistance and increases mechanical stability of the material, so it can be used in oral drug delivery [47]. Depending on the specific application of alginate hydrogel, other modifiers than those described above are used to improve its mechanical properties. For example, in bone engineering, hydroxyapatite [48], polycaprolactone [49], or biosilica [32] are used, while higher values of Young’s modulus are provided by the addition of poly(ethylene glycol) diacrylate and poly(vinyl alcohol) [36].

Another important issue for alginate hydrogel applications is its degradation over time. Drying of the hydrogel leads to a change in the geometric dimensions of the component [27,50]. This may result in limited functionality of such an element. Moreover, in the practice of cell culture cultivation on hydrogel substrates, the swelling ratio of the hydrogel is essential. Hydrogels with high swelling ratio can be used as an active matrix to absorb fluids and macromolecules [51]. It has been demonstrated that the swelling ratio of hydrogel decreases when the hydrogel component is exposed to drying [52], which may result in a lower absorption capacity. Because of these two issues, it is reasonable to use additives that reduce water loss through the hydrogel or minimize the effects of dehydration when the dried structure is rehydrated. During the drying process, the alginate network shrinks and collapses [53,54]. This is equivalent to increasing the density of cross-links and makes the dried alginate gel structure very dense [52]. Therefore, rehydration of the dried structure is difficult in pure water, but is possible in the presence of salt solutions [30,53]. The addition of other biopolymers can affect the drying process of the hydrogel by modifying the dry gel structure that is formed, and thus influence the subsequent swelling behavior of the material.

Gelatin is an additive that, in addition to improving the rheological properties of alginate hydrogel [38], promotes and improves the swelling of dried Ca(II). Cross-linked alginate structures with gelatin exhibit a higher swelling ratio than structures without it [52]. This is because the addition of gelatin leads to the formation of a less dense alginate hydrogel structure. Due to this, water penetration deep into the dry structure is facilitated, which makes the hydrogel return to its original size more efficiently during rehydration. On the other hand, gelatin is an additive that reduces the equilibrium swelling ratio of the hydrogel. This is caused by the fact that gelatin is a polymer that forms a gel network by itself [52]. Consequently, the gelatin network overlaps the alginate network, which is prevented from swelling further once a certain swelling ratio is achieved. Curti et al. [30] examined the dimensional stability of the scaffolds, also in aqueous solution. The scaffolds based on alginate and gelatin maintained their rectangular shape both when swollen and when dried. After reswelling the dried structures, they also concluded that increasing the gelatin content relative to alginate decreases the time required to rehydrate the structure. Alginate hydrogels with additive of gelatin are widely used as scaffolds for cell culture due to their very good swelling properties [55].

Another additive tested to minimize the effects of hydrogel drying is gum arabic. The mechanism of action of this substance is similar to gelatin. It leads to the formation of a less dense hydrogel structure after drying, which causes the component after rehydration to achieve a higher swelling ratio than that without gum arabic. In contrast to the hydrogel with gelatin, the addition of gum arabic does not reduce the equilibrium swelling ratio. This is due to the fact that gum arabic does not form a gel network by itself, and therefore does not reduce the swelling ratio of alginate hydrogel [52]. Alginate–gum arabic hydrogel has found application in oral drug delivery [22,23,24]. This combination provides protection of probiotic bacteria and drugs during drying and storage, and in the gastric tract [52]. Due to the frequent use of gum arabic as an additive for alginate hydrogels used in oral drug delivery, Chopra et al. [56] checked the swelling properties of dried hydrogel beads with ZnO nanoparticles inside. The analysis confirmed that addition of gum arabic leads to more efficient swelling during rehydration also with drugs inside.

Cellulose derivatives are additives widely used in the practice of alginate hydrogel formation [57,58,59]. One of them is carboxymethyl cellulose [49], the addition of which in appropriate amount makes the dried hydrogel sample swell faster after drying compared to pure alginate. However, it appears that an incorrect ratio makes the addition of CMC slow down the swelling process [53]. Another cellulose derivative that is used to create hydrogels is methyl cellulose (MC) [32,33]. Addition of methyl cellulose increases water retention capacity [60]. This is probably due to the fact that methyl cellulose may interfere with the intermolecular forces between alginate and water molecules. As a result, the attraction between those molecules may be stronger than in pure alginate hydrogels [60].

Additives used in the formation of hydrogels based on alginate allow the modification of the properties of this material, which allows for a very wide application of these hydrogels in medical practice. Nevertheless, it is reasonable to conduct further research on the search for additives that prevent water loss through hydrogel and minimize the effects of drying of alginate hydrogel. Research should focus on the proper selection of modifier concentration in relation to alginate that will ensure the best possible properties of hydrogel and, on the other hand, on the search for other, not yet used biopolymers or their derivatives.

### 3.2. Influence of Cross-Linking Parameters on Sodium Alginate Hydrogel Water Content

Cross-linking conditions have a huge impact on the water content in the hydrogel. Vargas et al. [23] noticed that cross-linking density affects water capacity due to the amount of defunded calcium ions in alginate backbones. Simoni [61], in her research on gelatin hydrogels, pointed out significant differences in their characteristics (swelling behavior and thermal characteristics) during drying by different methods—air and freeze drying. Moreover, hydrogel after freeze drying indicated microstructural modification; therefore, they were exposed to fragile fractures, whereas air-dried hydrogels maintained structural continuity and smooth surface. The present research also indicates that the cross-linking conditions influence the water content in the hydrogel to be evaporated. Both conditions, the cross-linking time and the concentration of the cross-linking agent (the amount of calcium ions that is incorporated into the alginate hydrogel structure), are important for the hydration of the hydrogel. The more calcium ions attach to the backbone of alginate, the less space is left for water molecules. On the other hand, the mechanical properties of a hydrogel are also dependent on the density of calcium ions in its structure. Higher concentration of Ca(II) ions can ensure hydrogel stability, and subsequently, less dehydration. In addition, the amount of calcium ions in the hydrogel structure also affects the color and transparency of the resulting hydrogels. The higher degree of cross-linking makes the hydrogel less transparent.

### 3.3. Mass Changes of Sodium Alginate Hydrogels in Different Environment Conditions

**Figure 3:**
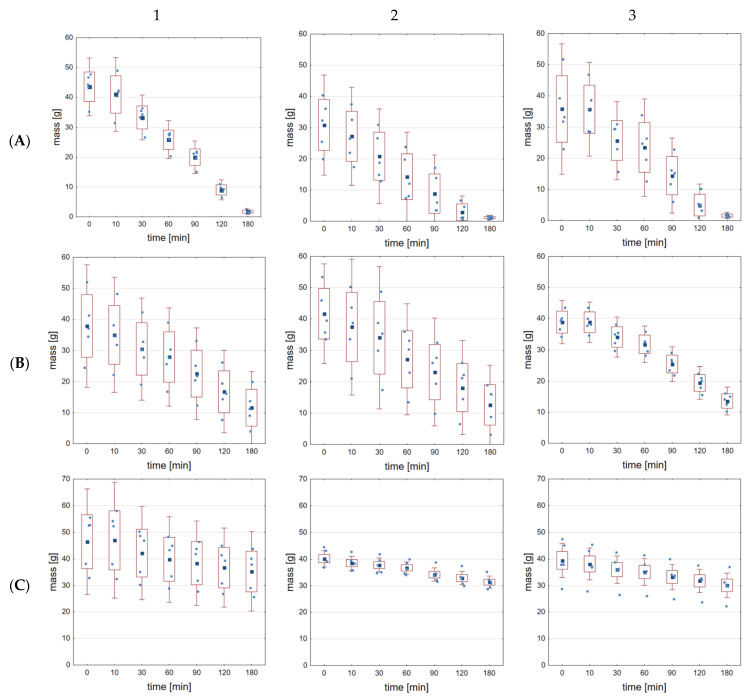
Mass changes during different types of drying process of alginate hydrogel ((**A**)—23 °C, 45% humidity, (**B**)—7 °C, 50% humidity, (**C**)—7 °C, 95% humidity) for 0.05 M of CaCl_2_ concentration in dependence on the cross-linking time (1–10 min, 2–20 min and 3–40 min).

The drying kinetics of alginate hydrogels cross-linked with calcium chloride according to the drying method are shown in Figure 3, and Supplementary Materials, Figures S1 and S2. The drying method depended on two environmental parameters: temperature and ambient humidity. It can be clearly observed that with increasing humidity and decreasing ambient temperature, the hydrogel mass decreased over time. For air-drying at 23 °C and 45% humidity (Figure 3A, and from Supplementary Materials Figures S1A and S2A), it can be seen that the mass reduction (water loss percentage) is the highest and, depending on the cross-linking time of the samples, that it reached a maximum of 96.15% (cross-linked for 20 min with calcium chloride at a concentration of 0.05 M) and a minimum of 82.44% (cross-linked for 40 min with calcium chloride at a concentration of 0.5 M). When the samples were dried at a lower temperature of 7 °C and ambient humidity of 50% (Figure 3B, and Supplementary Materials Figures S1B and S2B), the mass loss after 3 h of drying was 71.00% for 10 min of cross-linking time and at the lowest calcium chloride concentration (0.05 M), while the minimum value of cross-linking time (10 min) and the highest calcium chloride concentration (0.5 M) resulted in 45.24% water loss. By contrast, the lowest mass loss was found for the third drying method, 7 °C and 95% humidity (Figure 3C, and Supplementary Materials Figures S1C and S2C). When cross-linking sodium alginate with the middle concentration of CaCl_2_ (0.1 M) in the maximum time (40 min), the water loss was 36.34%, while for the middle cross-linking time (20 min) and the highest concentration of cross-linking agent (0.5 M), the water loss percentage was 18.06%. With the water amount lost, a change in color from bright yellow to orange was observed. A decrease in transparency was also noted, while the hydrogels after fabrication exhibited significant transparency; they became impermeable as the quantity of water lost. All the comparative data of water loss from alginate hydrogel are summarized in Table 2.

During the statistical analysis of the amount of water loss after the drying process in the first group (drying method A—23 °C, 45% humidity), statistically significant differences (for *p* < 0.05) were observed for 10 min of cross-linking between groups: 0.05 M and 0.5 M, but also 0.1 M and 0.5 M. For 20 min of cross-linking, statistically significant differences were noticed between all groups. For 40 min of cross-linking, statistically significant differences were observed only among 0.05 M and 0.5 M. In the second method of drying (B—7 °C, 50% humidity), statistically significant differences (for *p* < 0.05) were reported for 10 and 40 min of cross-linking between all groups. For 20 min of cross-linking, statistical differences were sighted between groups: 0.05 M and 0.5 M, as well as 0.1 M and 0.5 M. In the last drying method (C—7 °C, 95% humidity), statistically significant differences at *p* < 0.05 occurred for 10 min of cross-linking between groups: 0.05 M and 0.1 M, likewise 0.1 M and 0.5 M. For 20 min of cross-linking, statistically differences were found between groups: 0.05 M and 0.5 M, as well as 0.1 M and 0.5 M. For 40 min of cross-linking, statistically differences were noted between 0.1 M and 0.5 M.

It is clearly seen that environmental conditions, as well as technique of hydrogel cross-linking, have a significant impact during hydrogel drying. Humidity, as well as ambient temperature, can affect drying speed. Similarly, Kaklamani et al. [14] showed in their work the influence of high temperatures on drying kinetic of alginate hydrogel, cross-linked with calcium chloride. The research was conducted at 100, 150 and 200 °C in the restrained and unrestrained configurations. Restrained configuration includes local contact of a hot plate and samples attached by a stainless steel block, whereas unrestrained hydrogel showed a migration tendency across the hot plate. As the temperature increased, the drying speed also increased. On the other hand, a durable and robust structure was created, which improved the mechanical properties of the hydrogel.

A compromise between cross-linking time and cross-linking agent concentration must be found, due to the increased fragility of the hydrogel resulting from the high degree of cross-linking. As Aswathy et al. [6] wrote in their work, a high degree of cross-linking also reduces the swelling factor. It is an important factor in potential applications, e.g., in drug delivery systems.

### 3.4. Diameter Changes of Alginate Hydrogels in Different Environment Conditions

**Figure 4:**
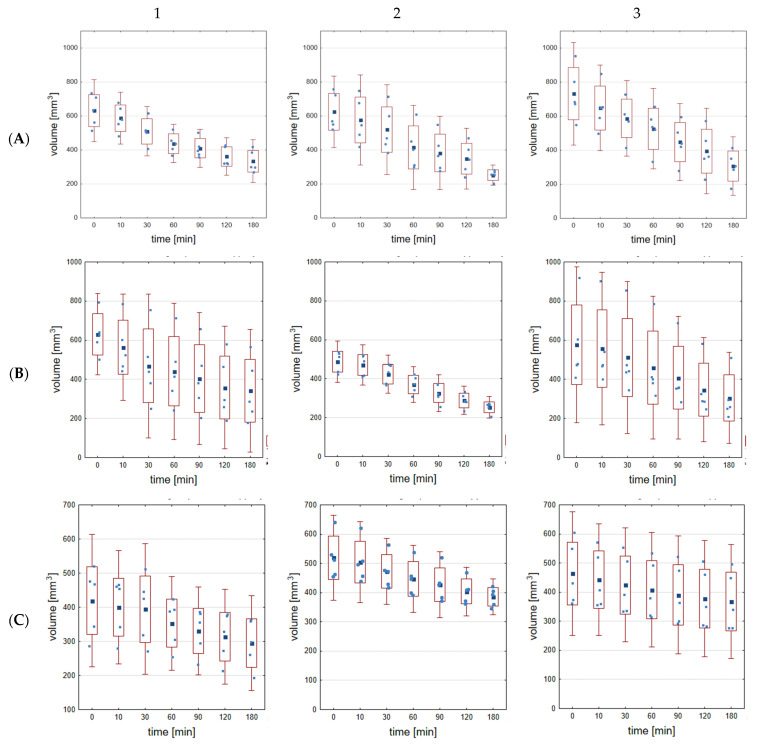
Dimension changes during different types of drying process of alginate hydrogel ((**A**)—23 °C, 45% humidity, (**B**)—7 °C, 50% humidity, (**C**)—7 °C, 95% humidity) for 0.05 M of CaCl_2_ concentration in dependence on cross-linking time (1–10 min, 2–20 min and 3–40 min).

In a manner equal to the hydrogel mass loss, the drying method also affected the differences in the dimensional and shape variability of the samples, as can be seen in Figure 4, and Supplementary Materials, Figures S3 and S4. Mass loss during the drying process under different conditions affected the shape change of the samples—their shrinkage. For air-drying at 23 °C and 45% humidity (Figure 4A, and Supplementary Materials Figures S3A and S4A), hydrogel cross-linked for 10 min with 0.5 M calcium chloride showed the highest dimensional and shape loss (64.57%). Hydrogel cross-linked for 40 min with 0.5 M calcium chloride exhibited the lowest dimensional and shape loss (33.63%). The second method of drying (Figure 4B, and Supplementary Materials, Figures S3B and S4B) at 7 °C and ambient humidity of 50% indicated that the lowest changes in dimensions (20.43%) occurred for 10 min of 0.5 M CaCl_2_ cross-linking and the highest (54.20%) for 10 min of 0.05 M CaCl_2_ cross-linking. Figure 4C, and Supplementary Materials, Figures S3C and S4C (7 °C and 95% humidity) show 29.53% (the highest change) loss of original dimensions for 10 min of 0.05 M CaCl_2_ cross-linking and 14.52% (the lowest change) loss of initial dimensions for 40 min of 0.5 M CaCl_2_ cross-linking. Table 3 presents the volume reduction percentage after different drying processes depending on the cross-linking conditions.

During the statistical analysis of the amount of volume reduction after the drying process in the first group (drying method A—23 °C, 45% humidity), statistically significant differences (for *p* < 0.05) were reported for 10 min of cross-linking between groups: 0.05 M and 0.5 M, likewise, also 0.1 M and 0.5 M. For 20 min of cross-linking, statistically significant differences were not observed. For 40 min of cross-linking, statistically significant differences were noticed among groups: 0.05 M and 0.5 M, as well as 0.1 M and 0.5 M. In the second method of drying (B—7 °C, 50% humidity), statistically significant differences (for *p* < 0.05) were noted for 10 min of cross-linking between 0.05 M and 0.1 M. For 20 min, of cross-linking statistical differences were observed between all groups. Statistical differences for 40 min of cross-linking were noticed only between 0.1 M and 0.5 M. In the last drying method (C—7 °C, 95% humidity), statistically significant differences at *p* < 0.05 appeared for 10 min of cross-linking only between groups: 0.05 M and 0.1 M. For 20 min of cross-linking, statistically differences were found between groups: 0.05 M and 0.5 M, likewise, 0.1 M and 0.5 M. For 40 min of cross-linking, statistically significant differences did not occur.

While there are applications where the shrinking capabilities of hydrogels can be utilized, far more applications require the dimensional retention behavior of hydrogels. The future direction will be concentrated on the development of hydrogels featuring anti-drying properties. Naranjo [62] prepared a hydrogel, which has a composition preserving water molecules in the polymer structure and can be used in applications such as artificial muscles; moreover, it has self-healing ability. Zhang et al. [18] investigated hydrogel (polyacrylic acid) with different drying methods (i.e., air-dried and using acetone solvent). It was assumed that hydrogel immersed in acetone during dehydration can create a flexible, self-healing structure, which, despite its shrinkage, is able to retain the shape after a few days of drying process. Wang et al. [63] carried out experiments on the influence of hydrogel on dimension retention of wool yarns. As a result, wool yarn treated with hydrogel indicated better dimension preservation and likewise, better strength, than uncoated yarn. 

**Figure 5:**
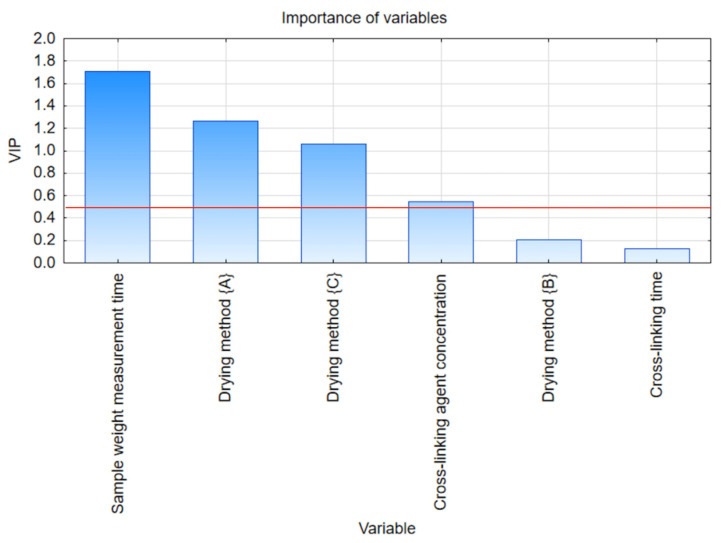
Variable Importance in Projection for weight changes during different types of drying process of alginate hydrogel.

Multivariate analysis was conducted by PLS in order to determine the importance of the variables influencing the process. The results of the analysis for weight changes during different types of drying process of alginate hydrogel are summarized in Figure 5. Significant features indicated by the PLS statistical method determined by the VIP indicator are included in Table 4. The importance trigger value was established at 0.5, according to the assumptions about the number of variables and the number of components.

After analyzing the statistical data, it can be seen that the most significant factor affecting the change in the weight of sodium alginate-based hydrogels is the sample weight measurement time, characterized by a VIP index value of 1.709. Subsequently, the importance of factors responsible for the hydrogel drying method are, successively, the {A} method with a VIP 1.265 indicator value and the {C} method with a VIP 1.061 indicator value. The last important factor influencing the process is the cross-linking agent concentration, whose VIP indicator reached 0.544.

**Figure 6:**
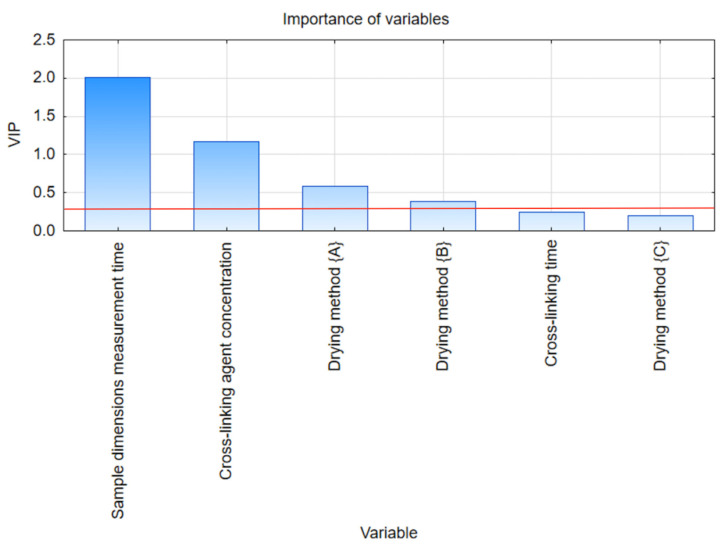
Variable Importance in Projection for dimension changes during different types of drying process of alginate hydrogel.

The results of the significance of the variables that influence the process of dimensional changes in sodium alginate hydrogel dried in different conditions are presented in Figure 6. Significant features identified by the PLS statistical method are defined by the VIP indicator, as is summarized in Table 5. In accordance with the presumptions regarding the quantity of variables and components, the importance trigger value was determined at 0.25.

The most significant factor affecting the change in the dimension of hydrogels based on sodium alginate is the sample weight measurement time, characterized by a VIP index value of 2.013. Furthermore, this indicator remains a higher value for changes in dimensions compared to changes in weight (1.709). Subsequently, the next important factor influencing the process is the cross-linking agent concentration, whose VIP indicator reached 1.164. This indicator also has a higher value for changes in dimensions than changes in hydrogel weight (0.544). The last variables with significant impact for the hydrogel drying method are, successively, the {A} method with a VIP 0.585 indicator value and the {B} method with a VIP 0.385 indicator value. In opposition to the change in weight of the hydrogel during drying, drying method {B} is a more important factor influencing the process during the analysis of dimensional change, whereas drying method {C} was more important during the second study.

The application of additive manufacturing technology in both medical and biomedical fields offers various and numerous advantages. In tissue engineering and regenerative medicine, its utilization for personalized production is crucial, particularly from the perspective of transplantation. However, due to significant constraints, like problems with printing the matrix with the given shape parameters and lack of preservation of the originally given geometry caused by dehydration of printing hydrogel structures, make additive technologies in medicine not yet fully usable. The worldwide interest in additive technologies brings hope for the creation of the best-suited implants and drug delivery systems. The development of additive manufacturing in biomedicine depends on the pace of development of other scientific branches, e.g., chemistry (researching new active substances and testing those already known), biomechanics (dealing with, for example, the distribution of stresses in the tested sample and the strength of materials), and information technology (improving computer systems and programs intended for designing and adapting 3D objects) [64].

## 4. Conclusions

The present paper evaluated the influence of cross-linking time and cross-linker concentration on sodium alginate hydrogel formation and on its shrinkage in different environmental conditions. Different temperatures (23 °C and 7 °C) and variable humidity (45%, 50% and 95%) were taken into account. The investigation was carried out in relation to the mass change during dehydration, and the dimensional and shape variation. The cross-linking time affected the change in the Ca(II) ions density in the hydrogel structure, and thus its water content. At the same time, higher cross-linking density determined better mechanical properties, which may improve structure maintenance during the evaporation of water from hydrogels. The study showed that depending on the storage of alginate hydrogels, the cross-linking time and calcium chloride concentration, the amount of water varied significantly. The best shape retention and the lowest water loss were observed for the lowest temperature (7 °C) at the highest ambient humidity (95%), at the middle cross-linking time (20 min) and the highest concentration of calcium chloride solution (0.5 M), and 18.25% water loss, respectively. By contrast, samples dried at a higher temperature (23 °C) with moisture content equal to 45% showed the highest water loss and shape variation. For 96.11% dehydration of hydrogel, the cross-linking time is 20 min and the cross-linking concentration is 0.05 M. These results clearly show that the best storage conditions that will ensure the least water loss from the hydrogel are low temperature (7 °C) and high ambient humidity (95%). This is the best way to store water-sensitive prints using AM technology immediately after the production process, before their utilization. The ability of shape retention is highly dependent on the concentration of cross-linking agent and the cross-linking time. The best initial shape representation is obtained for 0.5 M concentration of CaCl_2_ and 40 min cross-linking time (14.52% dimension changes). However, a compromise between cross-linking time and its concentration must be considered, as the amount of calcium ions contained in the structure affects the mechanical properties and can thus limit its applications. Developing a method to prevent dehydration of the hydrogel is quite a challenge, but improving methods to avoid water evaporation from the hydrogel will enable maintenance of dimensional and shape accuracy, which is very important for potential applications, especially in personalized medicine. Future studies can concentrate on increasing the range of temperature and humidity conditions in the direction of lower temperatures and high humidity. On the other hand, investigation may also focus on the use of additives that additionally provide better mechanical properties of hydrogels and also help to maintain the original shape.

## Figures and Tables

**Table 1:** Drying process parameters of alginate hydrogel cross-linked with calcium chloride.

Sample	Environmental Conditions		Cross-Linking Conditions	
	Temperature [°C]	Humidity [%]	CaCl_2_ Concentration [M]	Cross-Linking Time [min]
A/0.05 M/10 min	23	45	0.05	10
A/0.05 M/20 min	23	45	0.05	20
A/0.05 M/40 min	23	45	0.05	40
B/0.05 M/10 min	7	50	0.05	10
B/0.05 M/20 min	7	50	0.05	20
B/0.05 M/40 min	7	50	0.05	40
C/0.05 M/10 min	7	95	0.05	10
C/0.05 M/20 min	7	95	0.05	20
C/0.05 M/40 min	7	95	0.05	40
A/0.1 M/ 10 min	23	45	0.1	10
A/0.1 M/20 min	23	45	0.1	20
A/0.1 M/40 min	23	45	0.1	40
B/0.1 M/10 min	7	50	0.1	10
B/0.1 M/20 min	7	50	0.1	20
B/0.1 M/40 min	7	50	0.1	40
C/0.1 M/10 min	7	95	0.1	10
C/0.1 M/20 min	7	95	0.1	20
C/0.1 M/40 min	7	95	0.1	40
A/0.5 M/10 min	23	45	0.5	10
A/0.5 M/20 min	23	45	0.5	20
A/0.5 M/40 min	23	45	0.5	40
B/0.5 M/10 min	7	50	0.5	10
B/0.5 M/20 min	7	50	0.5	20
B/0.5 M/40 min	7	50	0.5	40
C/0.5 M/10 min	7	95	0.5	10
C/0.5 M/20 min	7	95	0.5	20
C/0.5 M/40 min	7	95	0.5	40

**Table 2:** Percentage amount of water loss after drying process.

Drying Method	Cross-Linking Time	Percentage of Water Lost [%] for Different Concentrations of CaCl_2_		
		0.05 M	0.1 M	0.5 M
A	10 min *	95.94 ± 0.80 ^a^	95.69 ± 0.53 ^b^	86.77 ± 0.83 ^ab^
	20 min *	96.15 ± 0.25 ^cd^	94.86 ± 0.43 ^ce^	82.55 ± 0.57 ^de^
	40 min **	95.56 ^f^	94.12	82.44 ^f^
B	10 min *	71.00 ± 8.33 ^ab^	54.19 ± 2.40 ^ac^	45.24 ± 0.90 ^abc^
	20 min *	70.88 ± 12.36 ^d^	65.68 ± 3.51 ^e^	52.93 ± 1.37 ^de^
	40 min *	65.25 ± 3.09 ^fg^	52.93 ± 1.79 ^fh^	60.59 ± 1.52 ^gh^
C	10 min *	23.81 ± 2.61 ^a^	29.55 ± 2.37 ^ab^	19.53 ± 3.30 ^b^
	20 min **	20.91 ^c^	21.57 ^d^	18.06 ^cd^
	40 min **	21.94	36.34 ^e^	20.00 ^e^

a–h—statistically significant differences for *p* < 0.05. * Tukey test (result presented as mean ± standard deviation. ** Kruskal–Wallis test (result presented as median).

**Table 3:** Percentage amount of volume reduction after drying process.

Drying Method	Cross-Linking Time	Percentage of Dimension Changes [%] for Different Concentrations of CaCl_2_		
		0.05 M	0.1 M	0.5 M
A	10 min *	47.21 ± 3.64 ^a^	43.24 ± 9.92 ^b^	64.57 ± 2.87 ^ab^
	20 min *	57.44 ± 5.18	49.38 ± 8.73	54.63 ± 4.92
	40 min **	56.90 ^c^	58.90 ^d^	33.63 ^cd^
B	10 min **	54.20 ^a^	29.69 ^a^	20.43
	20 min *	47.91 ± 3.74 ^bc^	35.84 ± 6.05 ^bd^	33.52 ± 6.93 ^cd^
	40 min *	47.50 ± 2.62	42.68 ± 5.36 ^e^	49.05 ± 1.81 ^e^
C	10 min *	29.53 ± 5.16 ^a^	26.23 ± 6.12 ^a^	24.59 ± 7.65
	20 min **	26.26 ^b^	25.71 ^c^	19.35 ^bc^
	40 min *	24.21 ± 3.22	16.76 ± 8.24	14.52 ± 3.28

a–e—statistically significant differences for *p* < 0.05. * Tukey test (result presented as mean ± standard deviation). ** Kruskal–Wallis test (result presented as median).

**Table 4:** Important features indicated by statistical method PLS and VIP scores for weight changes during different types of drying process of alginate hydrogel.

Variable	Variable Number	VIP	Importance
Sample weight measurement time	2	1.709	1
Drying method {A}	4	1.265	2
Drying method {C}	4	1.061	3
Cross-linking agent concentration	5	0.544	4
Drying method {B}	4	0.204	5
Cross-linking time	3	0.131	6

**Table 5:** Important features indicated by statistical method PLS and VIP scores for dimension changes during different types of drying process of alginate hydrogel.

Variable	Variable Number	VIP	Importance
Sample dimensions measurement time	2	2.013	1
Cross-linking agent concentration	5	1.164	2
Drying method {A}	4	0.585	3
Drying method {B}	4	0.385	4
Cross-linking time	3	0.247	5
Drying method {C}	4	0.200	6

## Supplementary Materials

The following supporting information can be downloaded at: https://www.mdpi.com/article/10.3390/gels9010063/s1, Figure S1: Mass changes during different type of drying process of alginate hydrogel (A—23 °C, 45% humidity, B—7 °C, 50% humidity, C—7 °C, 95% humidity) for 0.1 M of CaCl_2_ concentration in dependence on cross-linking time (1–10 min, 2–20 min and 3–40 min). Figure S2: Mass changes during different type of drying process of alginate hydrogel (A—23 °C, 45% humidity, B—7 °C, 50% humidity, C—7 °C, 95% humidity) for 0.5 M of CaCl_2_ concentration in dependence on cross-linking time (1–10 min, 2–20 min and 3–40 min). Figure S3: Dimensions changes during different type of drying process of alginate hydrogel (A—23 °C, 45% humidity, B—7 °C, 50% humidity, C—7 °C, 95% humidity) for 0.1 M of CaCl_2_ concentration in dependence on cross-linking time (1–10 min, 2–20 min and 3–40 min). Figure S4: Dimensions changes during different type of drying process of alginate hydrogel (A—23 °C, 45% humidity, B—7 °C, 50% humidity, C—7 °C, 95% humidity) for 0.5 M of CaCl_2_ concentration in dependence on cross-linking time (1–10 min, 2–20 min and 3–40 min).
