# Supplementary material for: Influence of Cross-Linking Conditions on Drying Kinetics of Alginate Hydrogel
Source: Gels. 2023 Jan 12;9(1):63. doi: 10.3390/gels9010063 (PMC9858758; doi:10.3390/gels9010063)
Supplement: Supplementary file 1 [file gels-09-00063-s001.zip › gels-2102434-supplementary.pdf]

Supplementary Materials

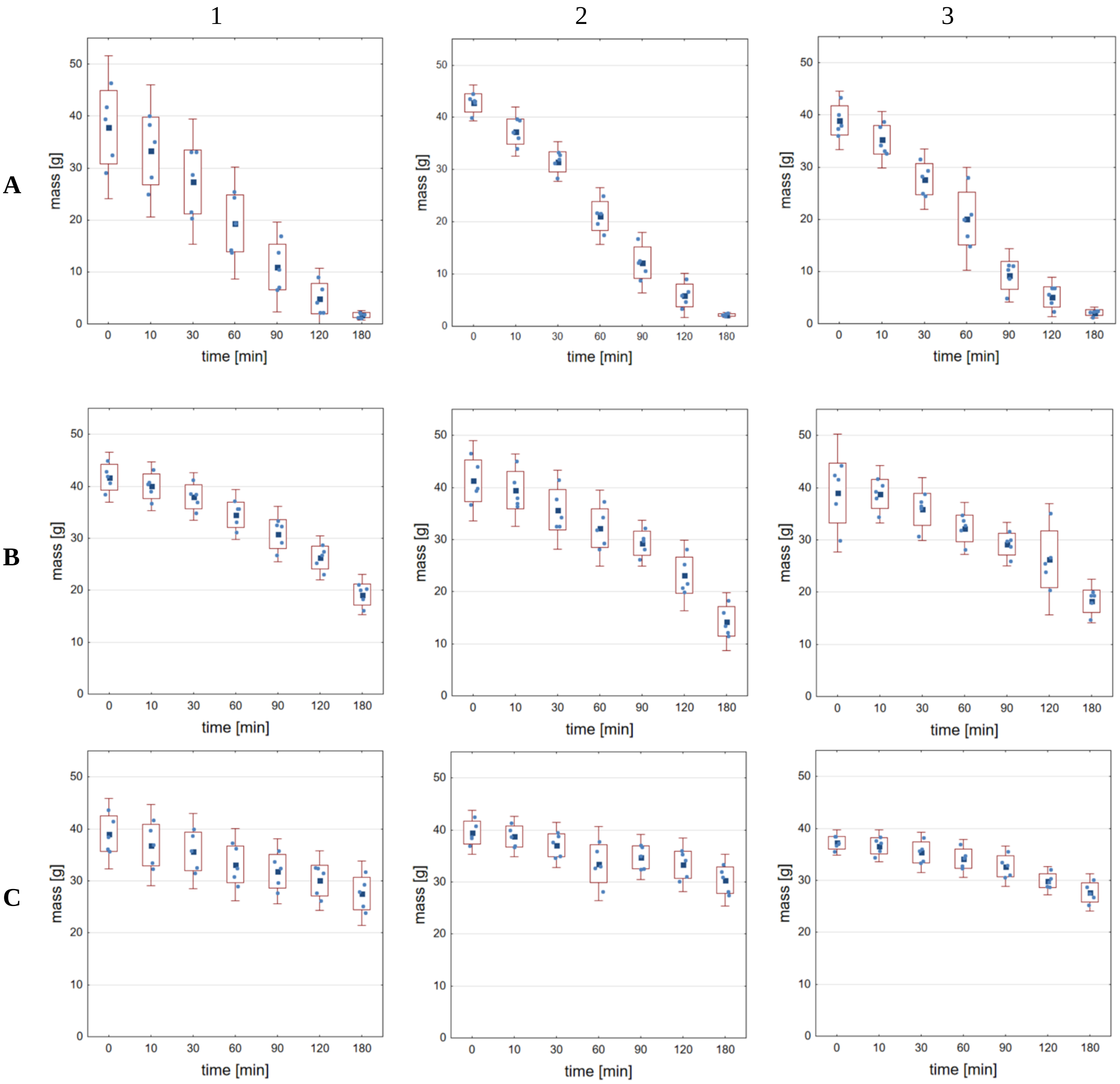

Figure S1. Mass changes during different type of drying process of alginate hydrogel ((**A**)–23°C, 45% humidity, (**B**)–7°C, 50% humidity, (**C**)–7°C, 95% humidity) for 0.1 M of $CaCl_2$ concentration in dependence on cross-linking time (1–10 min, 2–20 min and 3–40 min).

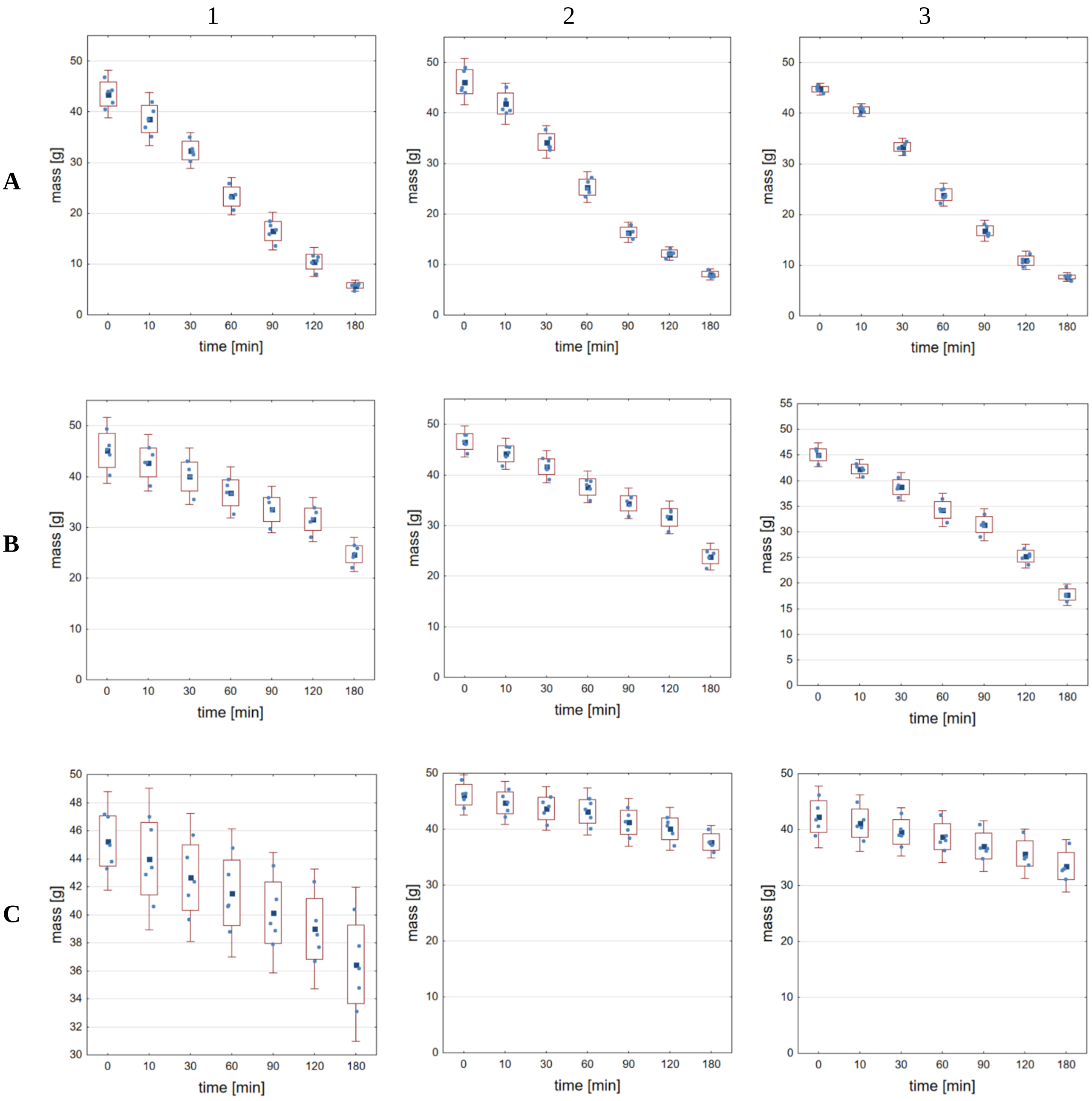

Figure S2. Mass changes during different type of drying process of alginate hydrogel ((**A**)–23°C, 45% humidity, (**B**)–7°C, 50% humidity, (**C**)–7°C, 95% humidity) for 0.5 M of $CaCl_2$ concentration in dependence on cross-linking time (1–10 min, 2–20 min and 3–40 min)

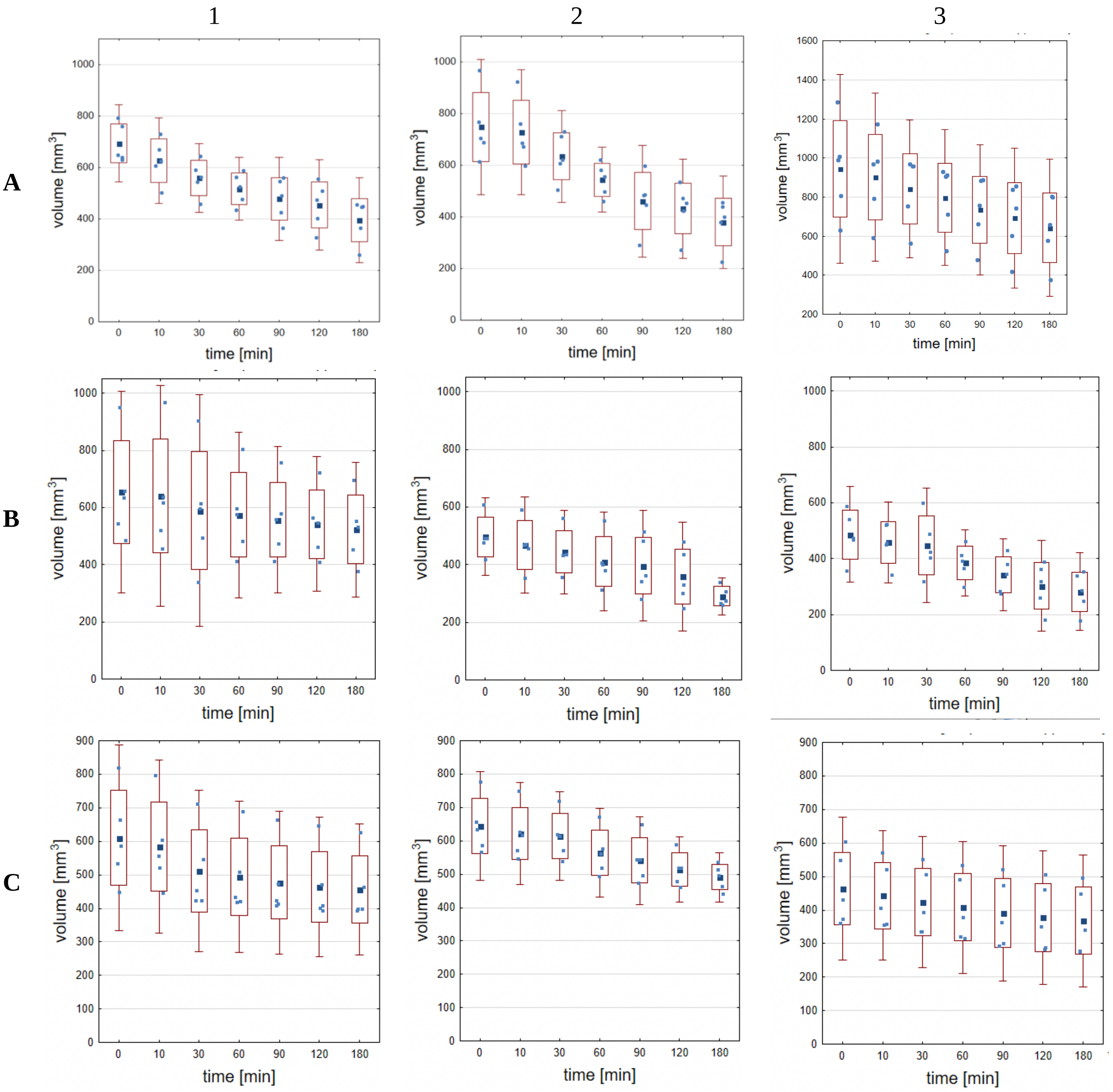

Figure S3. Dimensions changes during different type of drying process of alginate hydrogel ((**A**)–23°C, 45% humidity, (**B**)–7°C, 50% humidity, (**C**)–7°C, 95% humidity) for 0.1 M of $CaCl_2$ concentration in dependence on cross-linking time (1–10 min, 2–20 min and 3–40 min)

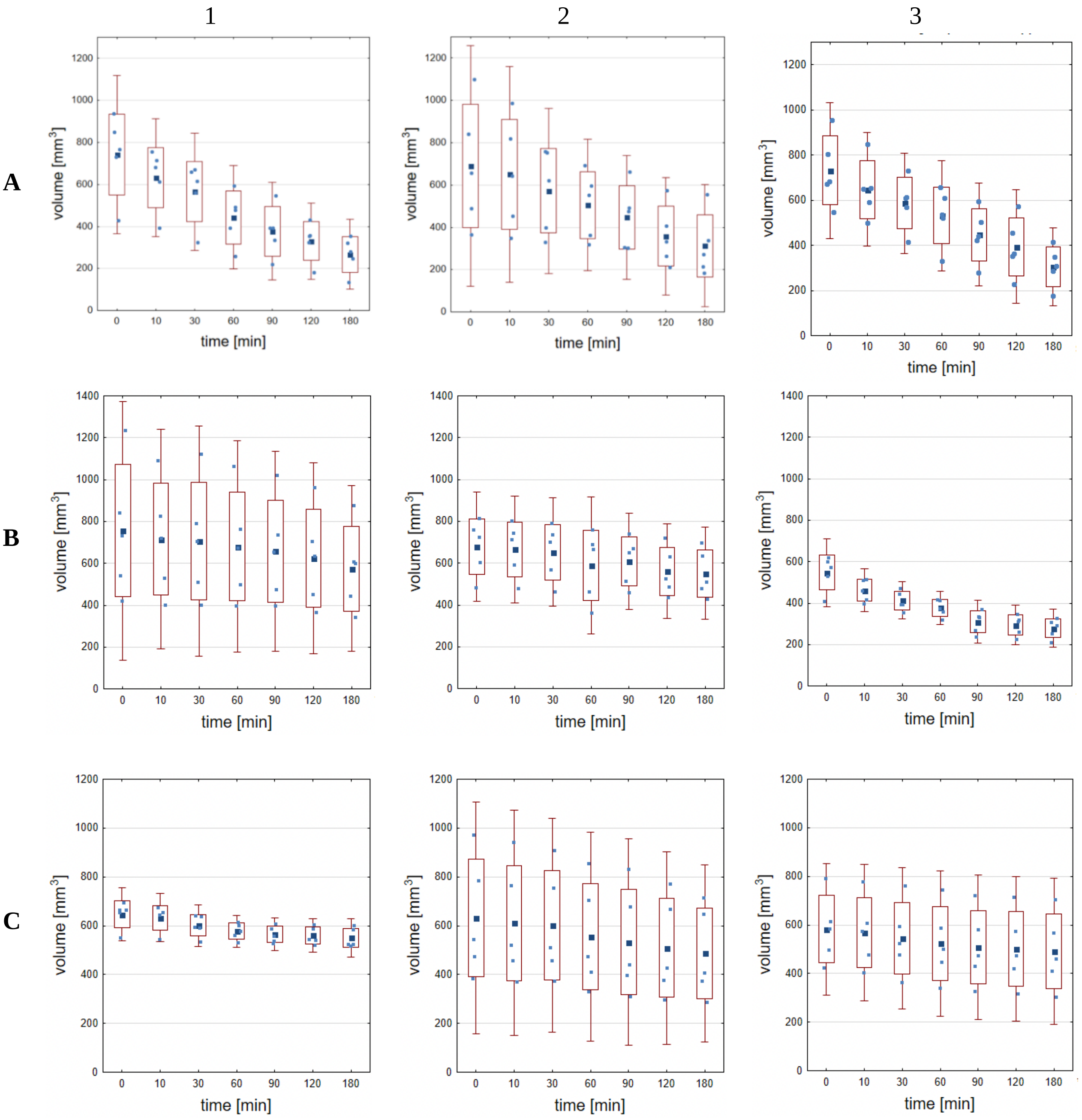

Figure S4. Dimensions changes during different type of drying process of alginate hydrogel ((**A**)–23°C, 45% humidity, (**B**)–7°C, 50% humidity, (**C**)–7°C, 95% humidity) for 0.5 M of $CaCl_2$ concentration in dependence on cross-linking time (1–10 min, 2–20 min and 3–40 min).
